# The complete mitochondrial genome of *Coccotorusbeijingensis* Lin et Li, 1990 (Coleoptera, Curculionidae, Curculioninae, Anthonomini) and its phylogenetic implications

**DOI:** 10.3897/BDJ.10.e95935

**Published:** 2022-12-09

**Authors:** Sai Jiang, Lina Jiang, Ran Li, Hui Gao, Aijing Zhang, Yurong Yan, Xinzhu Zou, Jixiao Wu, Shuying Xu, Xianfeng Yi, Yujian Li

**Affiliations:** 1 School of Life Sciences, Qufu Normal University, Qufu, China School of Life Sciences, Qufu Normal University Qufu China

**Keywords:** mitochondrial genome, *Coccotorusbeijingensis*, phylogenetic analysis

## Abstract

*Coccotorusbeijingensis* Lin et Li, 1990 belongs to Coleoptera, Curculionidae, Curculioninae, Anthonomini. It is a herbivorous insect that damages *Celtisbungeana* Blume (Ulmaceae) by affecting branch growth. The mitochondrial genome of *C.beijingensis* was sequenced and annotated to better identify *C.beijingensis* and related species. The total length of the *C.beijingensis* mitochondrial genome was 17,071 bp, contained 37 typical genes (13 protein-coding genes, two ribosomal RNA genes, 22 transfer RNA genes) and two control regions (total length: 2,292 bp). Mitochondrial genome composition, nucleotide composition and codon usage are similar to those of other sequenced Curculionidae mitogenomes. All protein-coding genes initiated with ATN and TTG codons and ended with TAA, TAG or incomplete stop codons (TA, T). In addition, analyses of pairwise genetic distances between individual PCGs in Curculionidae species showed that ATP8 was the least conserved gene, while COI was the most conserved. Twenty-one transfer RNAs had typical cloverleaf structures, while trnS1 lacked dihydrouridine (DHU) arms. ML and BI analyses, based on 13 PCGs and two rRNAs from ten species of Curculionidae, strongly support the relationships between *C.beijingensis* and species of the genus *Anthonomus*: ((*An.eugenii*+ *An.rubi*) + *C.beijingensis* + (*An.pomorum*+ *An.rectirostris*)) (BS = 100; PP = 1). Our phylogenetic analyses could mean that the genus *Coccotorus* should be sunk under *Anthonomus*, but more taxon sampling is needed to verify this result.

## Introduction

Mitochondria are important organelles involved in metabolism, apoptosis and life cycle of insect cells (Wolstenholme 1992). The mitochondrial genome has the advantages of maternal inheritance, stable conformation, low recombination rate, faster evolutionary rates than nuclear genes and no intron and has become an important research object in insect species identification, comparative genomics, phylogeny, population genetics and evolutionary biology (Wilson et al. 1985, Galtier et al. 2009). The insect mitochondrial genome is a double-linked loop of DNA (Avise 2009). The genome is 14–17 kb in length (Boore 1999, Cameron 2014). It consists of two ribosomal RNA genes (rRNAs), 22 transfer RNA genes (tRNAs), 13 protein-coding genes and two control regions (A + T-rich region), which contains some initiation sites for transcription and replication of the genome (Wolstenholme 1992, Boore 1999, Li et al. 2020). There are more genes encoded on the main strand (J) than the side strand (N) (Simon et al. 1994). With advances in high-throughput sequencing technologies reducing the cost of sequencing, the number of insect mitochondrial genomes sequenced has increased rapidly (Phillips et al. 2004).

*Coccotorusbeijingensis* Lin et Li, 1990 is widely distributed in northern China. It is harmful to the shoots of *Celtisbungeana* Blume (Urticales, Ulmaceae), causing tissue proliferation and formation of galls, affecting the growth of branches and even causing dead branches (Lin and Li 1990, Chen 1993). *C.beijingensis*'s only host plant is *Celtisbungeana* (Chen 1993). *Celtisbungeana* is tolerant of cold and is slow growing. *C.bungeana* is a precious garden-tree species (Cao and Gong 2021), used as a landscape plant (Liu 2016). *C.beijingensis* produce one generation a year in the Beijing area. The males begin entering the females' galls to mate in late March. The female adult begins to lay eggs in early April by biting a wound near the terminal bud of the new stem. The hatching larvae begin eating the plant tissue after ten days. It stimulated the plant tissue to proliferate and expand rapidly. In July, the galls had fully lignified. Adult weevils emerged in September after pupating in August. After emerging, each adult bit a circular opening at the front of the gall that had a diameter of about 2 nm. Lin concluded that this weevil was a monophagous pest (Lin and Li 1990, Li and Lin 1992).

The genus *Anthonomus* contains over 700 species, of which some are considered agricultural pests (Vossenberg et al. 2019, Zijp and Blommers 2002, Perkin et al. 2021, Revynthi et al. 2021, Tonina et al. 2021). There are only four known species of the genus *Coccotorus* in the world (Burke and Ahmad 1967), all of them from the Americas (Dietz 1891, Brown 2012). There has not been a molecular phylogenetic analyses that includes these two genera. To date, only four complete genome sequences of the tribe Anthonomini have been sequenced and annotated. The other mitochondrial genomes available for Anthonomini are incomplete or unclassified (https://www.ncbi.nlm.nih.gov/). In this study, we sequenced and annotated the mitochondrial genome of *C.beijingensis* and analysed its characteristics. In a previous study (Burke and Ahmad 1967), *Coccotorus* is considered a distinct genus, based on larval and pupal characters. In order to confirm the status and relationships of these two genera, we reconstructed the molecular phylogenetic relationships between *C.beijingensis* and several species in the genus *Anthonomus*. The molecular data provided in this study will contribute to the identification of *C.beijingensis* and its related species.

## Methods

### Sample collection and DNA extraction

Adult specimens of *Coccotorusbeijingensis* were collected from *Celtisbungeana* in Shimen Mountain Forest Park, Jining City, Shandong Province, China (35.785462°N，117.119296°E，April 2022). All fresh specimens were stored in 100% ethanol and stored in a -80℃ refrigerator in the laboratory of College of Life Sciences, Qufu Normal University. Adult specimen identification was based on morphological characteristics (Lin and Li 1990). Genomic DNA was extracted from 12 leg muscle tissues of two specimens using SanPrep DNA GelExtraction Kit (Sangon, Shanghai, China). A total of 74 female and 66 male specimens were kept in the College of Life Science, Qufu Normal University, Shandong Province.

### Mitogenome sequencing, assembly, annotation and bioinformatic analyses

Genomic DNA of all samples was sent to Personalbio Inc. (Shanghai, China) for library construction and next-generation sequencing (NGS). One library (Insert size of 400 bp) was prepared for each DNA sample using the TruSeqTM DNA Sample Prep Kit (Illumina, USA). All constructed libraries were then sequenced as 150 bp paired-end on a full run (2 × 150 PE) using the Illumina NovaSeq platform. Reads for species library have been deposited in the BioProject (PRJNA903367). The paired ends reads were *de novo* assembled using Novoplasty 3.7 (Dierckxsens et al. 2016). After trimming the adapters and removing short and low-quality reads, more than 4 GB (30−41 million reads) clean data for each sample were used in de novo assembly. About 2.5 Gb data were assembled into a complete circular mitogenome by DNASTAR 7.1 (Lasergene version 5.0) (Burland 2000) using the complete mitogenome of *Anthonomusrubi* (GenBank accession: NC 044714) as an initial seed. The mitogenome was annotated using MITOZ v.1.04 (Meng et al. 2019) and checked manually in Geneious v.8.1.3 (Kearse et al. 2012). The tRNA secondary structures were manually drawn using Adobe lllustrator CC2017, based on the MITOS Web Server predictions (Brent et al. 2013). The mitogenome map was drawn with the programme Organellar Genome DRAW (OGDRAW) (Lohse et al. 2013). Bioinformatic analyses, including nucleotide composition, composition skew, codon usage of PCGs, relative synonymous codon usage (RSCU) and mitogenomic organisation tables were conducted using PhyloSuite v.1.2.2 (Zhang et al. 2019).

### Molecular phylogenetic analysis

A total of eleven mitogenomes from Curculionidae were used for the phylogenetic analyses (Table 1). We used all mitochondrial genome data available for the genus *Anthonomus* to test the placement of *C.beijingensis*. Two species of *Aclees*, subfamily Molytinae, were considered as outgroups. Nucleotide sequences (without stop codons) for the 13 PCGs were aligned using MAFFT v.7 (Katoh et al. 2002), with the G-INS-i (accurate) strategy and codon alignment mode (Code table: Invertebrate mitochondrial genetic codon). The rRNAs genes (rrnL and rrnS) were aligned using MAFFT v.7 (Katoh et al. 2002) with the Q-INS-I algorithm (which takes account of the secondary structure of rRNA genes). Ambiguously-aligned areas were removed using Gblocks 0.91b (Talavera and Castresana 2007). Gene alignments were concatenated using PhyloSuite v.1.2.2 (Zhang et al. 2019). Partitioning scheme and nucleotide substitution models for Maximum Likelihood (ML) and Bayesian Inference (BI) phylogenetic analyses were selected with ModelFinder (Kalyaanamoorthy et al. 2017) using the Bayesian Information Criterion (BIC). ML analyses were reconstructed by IQ-TREE v.1.6.3 (Nguyen et al. 2015) under the ultrafast bootstrap (UFB) approximation approach with 5,000 replicates. BI analysis was performed using MrBayes v.3.2.7a (Ronquist et al. 2012) in the CIPRES Science Gateway (Miller et al. 2010) with four chains (one cold chain and three hot chains). Two independent runs of 2,000,000 generations were carried out with sampling every 1,000 generations. The first 25% of trees were discarded as burn-in. After the average standard deviation of split frequencies fell below 0.01, stationarity was assumed. The resulting phylogenetic trees were visualised in FigTree 1.4.0 (https://tree.bio.ed.ac.uk/software/figtree/).

## Results and discussion

### Mitogenome organisation and nucleotide composition

**Figure 1.**
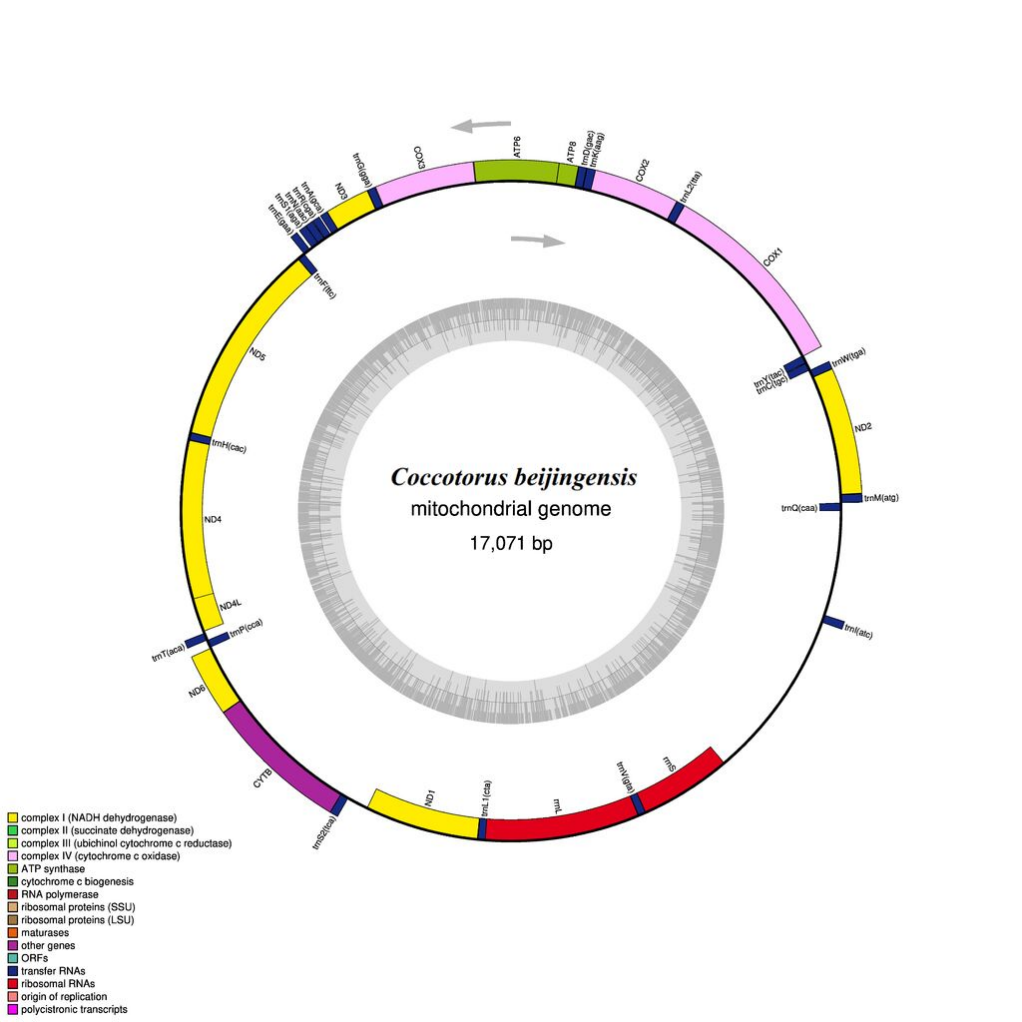
Circular map of the mitogenome of *C.beijingensis*. The outer circle shows the gene map of *C.beijingensis* and the genes outside the map are coded on the major strand (J-strand), whereas the genes on the inside of the map are coded on the minor strand (N-strand). Genes are represented by different colour blocks.

The mitochondrial genome of *Coccotorusbeijingensis* was a double-linked loop DNA molecule, containing 37 classic mitochondrial genes (13 PCGs, 22 tRNA and 2 rRNA) and two control regions (Table 2, Fig. 1).

The mitochondrial genome length measured in this study was 17,071 bp and the change in the size of the control region was the main source of the change in the mitochondrial genome length (Table 2). The mitochondrial genome sequence of *C.beijingensis* was consistent with that of other sequenced species in the Curculionidae. Nucleotide overlap was found in 13 pairs of genes, amongst which cox1 and trnL2 had the longest nucleotide overlap (8 bp). In addition, 11 pairs of genes had intergenic sequences, amongst which the longest spacer sequence was between trnS2 and nad1 (174 bp).

The mitochondrial genome of *Coccotorusbeijingensis* showed a strong AT bias in nucleotide content, with 73.6% AT content in the whole genome, the lowest AT content in PCGs (72.4%) and the highest AT content in tRNAs (76.8%) (Table 3). AT-skew and GC-skew of *C.beijingensis* mitochondrial genome were 0.043 and -0.159, respectively. In the mitochondrial genomes of selected species, the AT-skew of PCGs (-0.149 to -0.129) and rRNAs (-0.076 to -0.021) of each species were negative and the AT-skew of tRNAs (0.019 to 0.052) was positive. In the mitochondrial genomes of selected species, the GC-skew of PCGs (-0.94 to -0.021) of each species was negative and the GC-skew of tRNAs (0.084 to 0.121) and rRNAs (0.305 to 0.397) were positive (Table 3). Most of the above data of *C.beijingensis* were between the maximum and minimum values of each indicator for the eleven incuded species and only the GC-skew value of tRNAs was outside the range (0.277) (Table 3).

### Protein-coding genes

**Figure 2.**
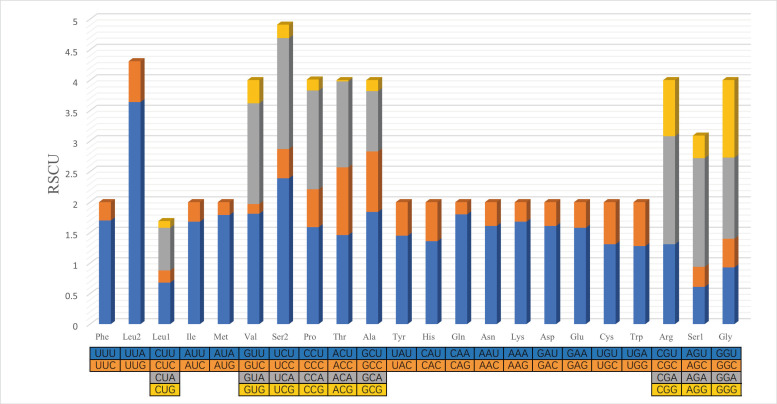
Relative synonymous codon usage (RSCU) of the mitogenome of *C.beijingensis*. The stop codons are not shown.

**Figure 3.**
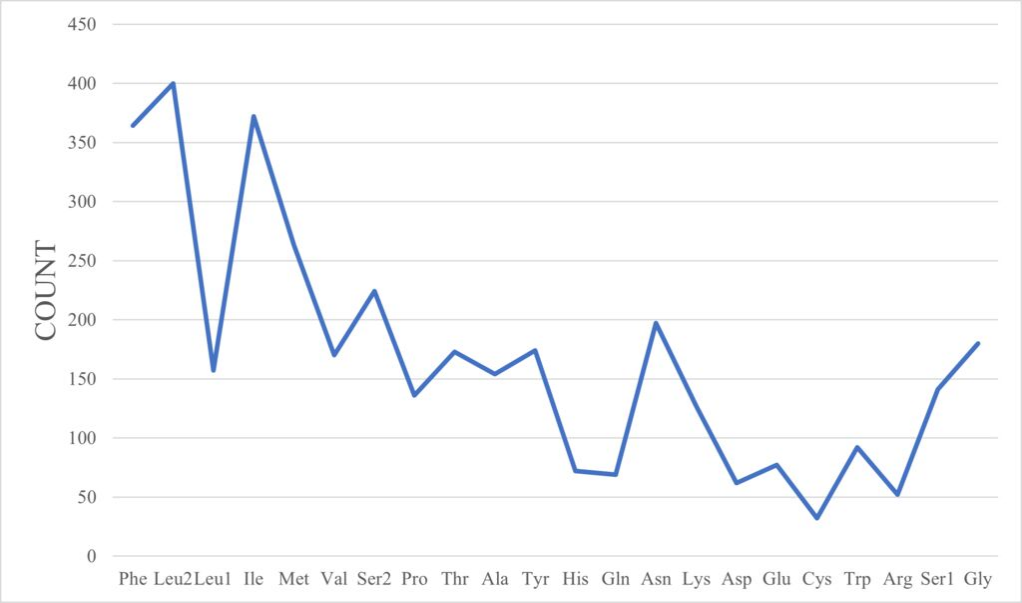
Statistics of amino acids encoded by protein-coding genes.

The total size of the 13 PCGs of *C.beijingensis* was 11,095 bp, accounting for 64.99% of the entire mitochondrial genome. Most of the 13 PCGs (COⅠ-Ⅲ, ND2-6, ND4L, ATP6 and ATP8) used ATN (ATA\ATT\ATG) as the start codon and only ND1 used TTG as the start codon. All PCGs stopped with TAA/G or their incomplete form T-. The incomplete termination codon single T- can be completed by post-transcriptional polyadenylation (Ojala et al. 1981). The relative synonymous codon usage (RSCU) of *C.beijingensis* mitogenome is presented in Fig. 2. The most commonly used codon was UUA-Leu, followed by UCU-Ser2 and GCU-Ala. Fig. 3 shows that Leu is the most commonly used amino acid, followed by Ile and Phe.

**Figure 4.**
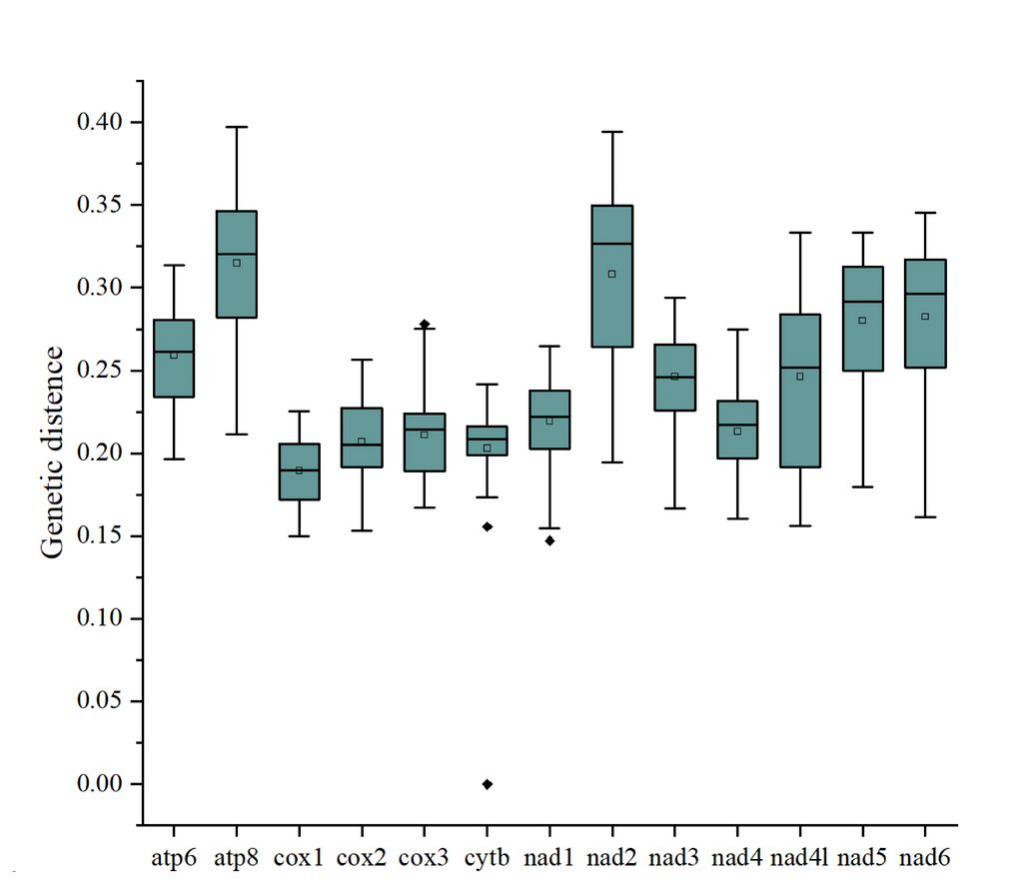
Genetic distances amongst individual PCGs. Each boxplot represents the P-distance for 13 individual genes in eleven species in Curculionidae. The box plot presents the minimum and maximum values at the ends of the whiskers, the 25^th^ and 75^th^ percentiles at the ends of a box, the median as a horizontal line in the box at the 50^th^ percentile value, hollow square represent the average and solid diamond as outliers.

Pairwise genetic distances in single PCG amongst eleven species in Curculionidae are shown in Fig. 4. ATP8 was the least conserved gene (average 0.32, range 0.29 to 0.33). COⅠ was the most conserved gene (average 0.19, range 0.17 to 0.20) and thus has been widely used as a molecular marker to explore phylogenetic relationship and population genetic diversity (Fig. 4).

### Transfer and ribosomal RNA genes

**Figure 5.**
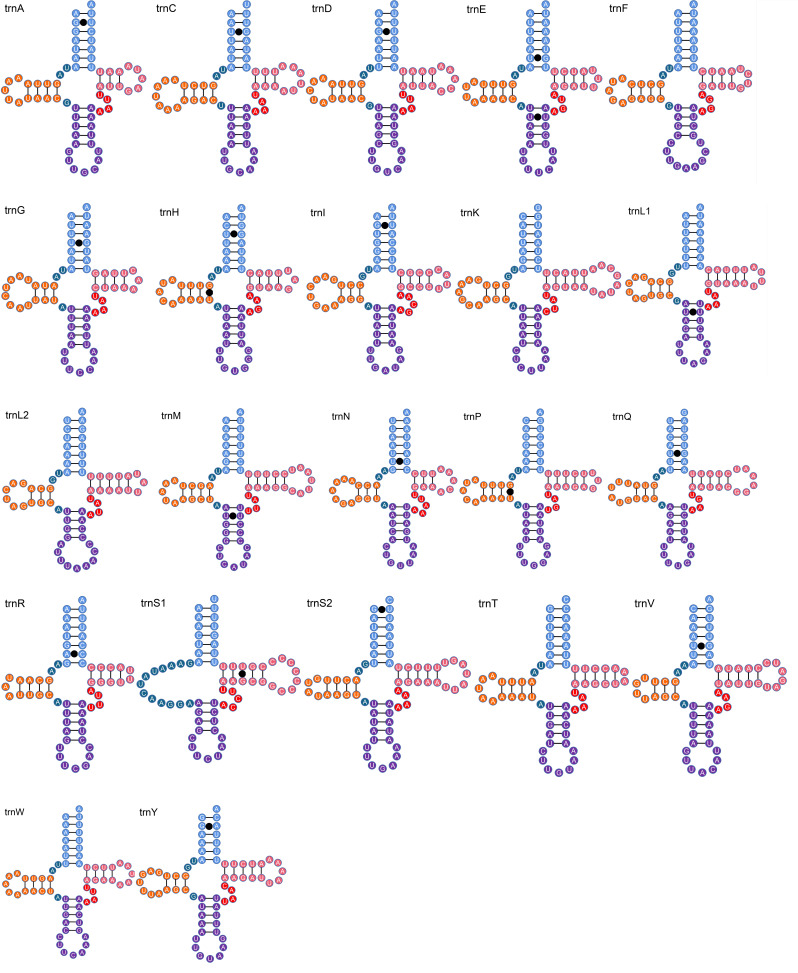
Secondary structures of 22 tRNAs in the mitogenome of *C.beijingensis*. Lines (-) indicate Watson-Crick base pairings, whereas solid black circles indicate unmatched base pairings.

*Coccotorusbeijingensis* had 22 typical tRNAs, ranging in length from 60 bp (trnR) to 70 bp (trnK) (Table 2). The AT content of tRNAs was 76.8% (Table 3). With the exception of trnS1, most tRNAs had a clover leaf secondary structure in which the dihydrouridine (DHU) arm becomes a simple loop (Fig. 5). This feature is common in metazoan mitogenomes (Garey and Wolstenholme 1989). *Coccotorusbeijingensis*'s rrnL (1,292 bp) was located between trnL1 and trnV, while rrnS (770 bp) was located between trnV and the control region. The location was similar to that of other species of the Curculionidae family (Hu et al. 2021). Amongst the 22 tRNAs, there were 19 unmatched base pairs, which belonged to four types (GU\UU\AC\GA) (Fig. 5).

### Control region

The control region regulates the replication and transcription of mtDNA (Clayton 1991). In this study, the control regions of *C.beijingensis* were located in two parts (control region 1 and control region 2) and trnI was located between the two control regions. This peculiar structure of the non-coding region may be a derived character of Curculionoidea (Narakusumo et al. 2020). The CR1 (control region 1) was located between rrnS and trnI. CR2 (control region 2) was located between trnI and trnQ. The length and AT content of CR1 (1,424 bp) were longer than CR2 (868 bp) (Table 2).

### Phylogenetic relationships

**Figure 6.**
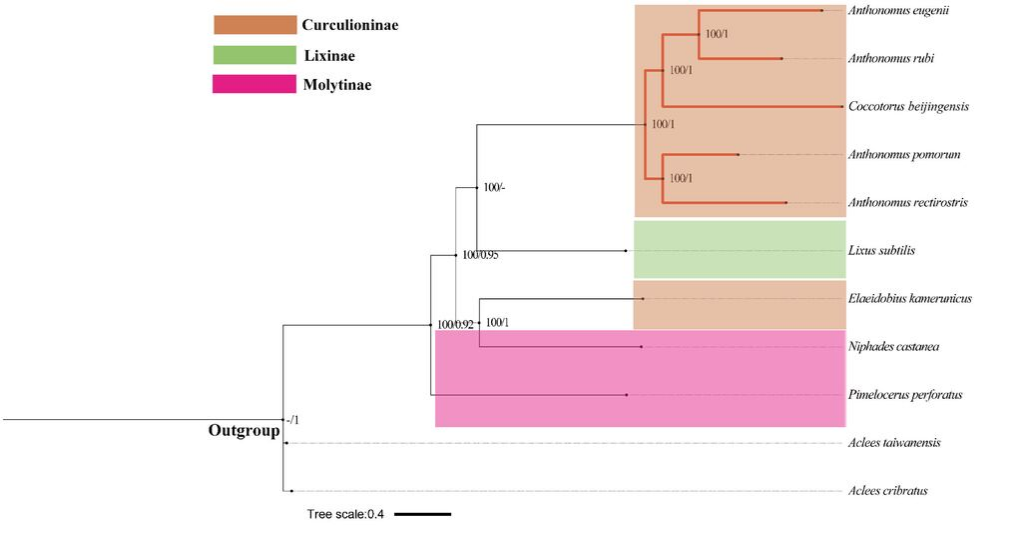
ML and BI phylogenetic trees for *C.beijingensis*, based on the nucleotide sequence data of 13 PCGs and two rRNAs from *C.beijingensis* and other ten species belonging to three subfamilies in the family of Curculionidae. Bootstrap support values (BS) and Bayesian posterior probabilities (PP) are indicated on the branch.

Based on ML and BI analyses of 13 PCGs and rRNAs nucleotide data, we reconstructed the phylogenetic relationships of 11 Curculionidae species, including *C.beijingensis*. Both phylogenetic trees had congruent topological structure and all branches were strongly supported (Fig. 6). In addition, it was found in our study that *C.beijingensis* clusters with the four Anthonomus species included in the analyses, forming a monophyletic group with the highhest support (BS = 100; PP = 1). Through phylogenetic analysis, we obtained the following genetic relationships within Anthonomini: ((*An.eugenii* + *An.rubi*) + *C.beijingensis* + (*An.pomorum* + *An.rectirostris*)). Our phylogenetic analyses could mean that the species *Coccotorusbeijingensis* should be sunk under the genus *Anthonomus*. This result does not agree with that these two taxa should be distinct genera (Burke and Ahmad 1967). However, since the genus *Anthonomus* contains over 700 species, additional taxon sampling is needed to verify this result.

## Data Resources

Reads for species library have been deposited in the BioProject (PRJNA903367).

### Resource 1

Download URL: https://www.ncbi.nlm.nih.gov/nuccore/ON808615

Resource identifier: ON808615

Data format : FASTQ

## Figures and Tables

**Table 1.** Mitogenomes of the 11 Curculionidae taxa used in this study.

Subfamily	Species	GenBank Accession number	Length (bp)	Reference
Curculioninae	*Anthonomuseugenii*	NC044711	17,257	Vossenberg et al. (2019)
	*Anthonomuspomorum*	NC044712	17,093	
	*Anthonomusrectirostris*	NC044713	17,676	
	*Anthonomusrubi*	NC044714	17,476	
	*Elaeidobiuskamerunicus*	NC049880	17,729	Apriyanto and Tambunan (2020)
	*Coccotorusbeijingensis*	ON808615	17,071	This study
Lixinae	*Lixussubtilis*	MW413392	15,223	Xing et al. (2021)
Molytinae	*Niphadescastanea*	MT232762	17,494	Unpublished
	*Acleestaiwanensis*	MZ305480	17,435	Unpublished
	*Acleescribratus*	NC051548	17,329	Wang et al. (2020)
	*Pimelocerusperforatus*	NC053826	15,977	Unpublished

**Table 2.** Mitogenomic organisation of *C.beijingensis*.

**Gene**	**Location**		**Size (bp)**	**IN**	**Anticodon**	**Codon**		**Strand**
	**From**	**To**				**Start**	**Stop**	
trnQ	1	67	67	—	CAA	—	—	-
trnM	67	135	69	-1	ATG	—	—	+
ND2	136	1134	981	—	—	ATA	TAA	+
trnW	1133	1197	65	-2	TGA	—	—	+
trnC	1197	1262	66	-1	TGC	—	—	-
trnY	1265	1331	67	2	TAC	—	—	-
COⅠ	1324	2866	1543	-8	—	ATT	T	+
trnL2	2867	2930	64	—	TTA	--	--	+
COⅡ	2931	3609	679	—	—	ATT	T	+
trnK	3606	3675	70	-5	AAG	—	—	+
trnD	3676	3740	65	—	GAC	—	—	+
ATP8	3741	3896	156	—	—	ATT	TAA	+
ATP6	3893	4558	666	-4	—	ATA	TAA	+
COⅢ	4561	5346	786	2	—	ATA	TAA	+
trnG	5349	5412	64	2	GGA	—	—	+
ND3	5413	5766	354	—	—	ATT	TAG	+
trnA	5765	5827	63	-2	GCA	—	—	+
trnR	5838	5897	60	10	CGA	—	—	+
trnN	5900	5963	64	2	AAC	—	—	+
trnS1	5961	6026	66	-3	AGA	—	—	+
trnE	6048	6109	62	21	GAA	—	—	+
trnF	6108	6171	64	-2	TTC	—	—	-
ND5	6172	7882	1711	—	—	ATT	T	-
trnH	7883	7945	63	—	CAC	—	—	-
ND4	7946	9272	1327	—	—	ATG	T	-
ND4L	9266	9550	285	-7	—	ATA	TAG	-
trnT	9564	9627	64	13	ACA	—	—	+
trnP	9628	9692	65	—	CCA	—	—	-
ND6	9698	10195	498	5	—	ATA	TAA	+
Cytb	10196	11335	1140	—	—	ATG	TAG	+
trnS2	11334	11401	68	-2	TCA	—	—	+
ND1	11576	12526	951	174	—	TTG	TAG	-
trnL1	12528	12592	65	1	CTA	—	—	-
rrnL	12588	13879	1292	-5	—	—	—	-
trnV	13880	13944	65	—	GTA	—	—	-
rrnS	13943	14712	770	-2	—	—	—	-
CR1	14713	16136	1424	—	—	—	—	+
trnI	16137	16202	66	—	ATC	—	—	+
CR2	16203	17071	868	—	—	—	—	+

**Table 3.** Base composition and skewness of mitogenomes of *N.castanea, L.subtilis, Acleestaiwanensis, Anthonomuseugenii, An.pomorum, An.rectirostris, An.rubi, E.kamerunicus, Ac.cribratus, P.perforatus* and *C.beijingensis*.

	A+T％				AT-skew				GC-skew			
species	All	PCGs	tRNAs	rRNAs	All	PCGs	tRNAs	rRNAs	All	PCGs	tRNAs	rRNAs
*N.castanea*	76.7	75.7	77.7	79.8	0.025	-0.149	0.022	-0.035	-0.204	-0.021	0.108	0.343
*L.subtilis*	75.7	75.1	76.9	79.3	0.062	-0.137	0.03	-0.041	-0.209	-0.036	0.104	0.362
*Ac.taiwanensis*	75.5	74.4	78.1	79.1	0.044	-0.145	0.045	-0.029	-0.245	-0.047	0.105	0.397
*An.eugenii*	72.5	71	75.1	76.4	0.054	-0.138	0.046	-0.058	-0.19	-0.083	0.092	0.313
*An.pomorum*	73.7	72.9	74.6	76.8	0.04	-0.133	0.052	-0.039	-0.178	-0.055	0.084	0.305
*An.rectirostris*	74.6	73.6	74.6	77.9	0.043	-0.13	0.043	-0.042	-0.205	-0.072	0.091	0.33
*An.rubi*	74.5	73.3	76.5	76.6	0.046	-0.141	0.022	-0.05	-0.201	-0.094	0.09	0.333
*E.kamerunicus*	73.5	72.1	77.1	77.7	0.085	-0.129	0.019	-0.076	-0.224	-0.069	0.109	0.318
*Ac.cribratus*	75.8	74.7	78	79.6	0.042	-0.143	0.046	-0.025	-0.247	-0.059	0.109	0.382
*P.perforatus*	75.5	74.2	78.5	79.3	0.038	-0.143	0.03	-0.021	-0.238	-0.042	0.121	0.333
*C.beijingensis*	73.6	72.4	76.8	77.7	0.043	-0.138	0.052	-0.022	-0.159	-0.055	0.095	0.277
